# The Role of Angiogenesis in Haemophilic Arthropathy: Where Do We Stand and Where Are We Going?

**DOI:** 10.4274/tjh.2016.0031

**Published:** 2016-05-16

**Authors:** Alexandra Agapidou, Thomas Stavrakis, Efthymia Vlachaki, Panagiotis Anagnostis, Sophia Vakalopoulou

**Affiliations:** 1 Aristotle University, Hippokration Hospital, Second Propaedeutic Department of Internal Medicine, Thessaloniki, Greece; 2 Aristotle University, Hippokration Hospital, Second Department of Internal Medicine, Thessaloniki, Greece

**Keywords:** Angiogenesis, Haemophilic arthropathy, Vascular endothelial growth factor, Haemophilia

## Abstract

Haemophilia is an inherited bleeding disorder that can lead to degenerative joint arthropathy due to recurrent bleeding episodes affecting the musculoskeletal system of the patient. The cause of bleeding can be either traumatic or spontaneous. The pathogenesis of haemophilic arthropathy is unclear as many factors like iron, inflammatory cytokines, and angiogenic factors contribute to this process. Blood into joints can deteriorate the bone to such an extent that the patient experiences pain, reduction of the range of movement, and deformity of the joint, conditions that could have a great impact on quality of life. Over the years, management of haemophilic arthropathy has changed. Nowadays, early diagnosis with high resolution imaging like magnetic resonance imaging along with application of prophylaxis regimens can reduce the extent of damage to the joints. However, not all haemophilia patients have access to these interventions as cost may be prohibitive for some of them. The need for new, easy, and cost-effective strategies with the ability to identify early changes could be beneficial and could make a difference in the management of haemophilic arthropathy. Understanding the mechanism of processes like angiogenesis in the mechanism of developing arthropathy could be innovative for these patients and could help in the detection of new early diagnostic and therapeutic markers.

## Abstract (tr)

Hemofili, hastanın kas ve iskelet sistemini etkileyen, tekrarlayan kanama atakları ile dejeneratif eklem artropatisine neden olan, kalıtsal bir kanama bozukluğudur. Kanama travma sonrasında ya da kendiliğinden olabilir. Hemofilik artropatinin patogenezi kesin bilinmemekle birlikte, demir, yangı sitokinleri ve anjiyogenik faktörlerin sürece katkıları vardır. Eklem içine kanama kemiği bir düzeye kadar bozabilir ve hasta, yaşam kalitesi üzerine büyük bir etkisi olan ağrı, hareket kısıtlılığı ve eklem deformitesi gibi durumları yaşar. Yıllar içerisinde hemofilik artropatinin yönetiminde değişiklikler olmuştur. Günümüzde manyetik rezonans görüntüleme gibi yüksek çözünürlüklü görüntüleme yöntemleri ile erken tanı ve profilaksi rejimlerinin kullanılması eklemlerdeki harabiyetin derecesini azaltmaktadır. Ancak bunların ücretleri bazı hastalar açısından sınırlayıcı olabileceğinden, tüm hastalar bu müdahalelere ulaşamaz. Erken dönemdeki değişiklikleri tespit edebilen yeni, kolay ve maliyet etkin stratejiler yararlı olabilir ve hemofilik artropatinin yönetiminde bir değişiklik yapabilir. Artropati gelişim mekanizmalarından anjiyogenez gibi süreçlerin mekanizmasının anlaşılması bu hastalar için bir yenilik olabilir ve yeni erken tanısal ve terapötik belirteçlerin bulunmasına yardımcı olabilir.

## INTRODUCTION

Haemophilia A and B are X-linked inherited disorders respectively caused by the deficiency of coagulation factor VIII or IX [1]. Lack of those clotting factors (CFs) leads to an increased tendency to bleed at various intensities, according to the percentage of the missing CF. The system that is mainly affected by these recurrent bleeding episodes is the musculoskeletal system. Repeated joint bleeds can cause progressive destruction of the cartilage, resulting in a decreased range of motion due to pain and stiffness. This condition is known as haemophilic arthropathy or haemophilic joint disease (HJD) and it has a progressively negative impact on patients’ quality of life. Haemophilia is found to be associated with decreased bone mass in both adults and children [2]. Haemarthrosis, formed after repeated joint bleeds, could be prevented by providing prophylaxis to these patients by means of administering the missing CF from an early age and in a standard regimen. However, this requires good venous access and skills from the patient’s point of view along with highly specialised and properly organised structures from the health provider’s side. If a patient cannot receive prophylactic treatment, there are various other ways of chronic joint pain relief, like applying interventions such as synovectomy and arthroplasty. These are invasive surgical procedures that may be frustrating for the patient. It is important to realise that the mechanism underlying progressive haemophilic arthropathy is multifactorial and still remains unclear. Availability of an easy, quick, and low-cost test with high specificity for diagnosing HJD would be beneficial for both patients and health providers. Use of the Fracture Risk Assessment Tool for assessing fracture risk, regular bone mineral density assessment, and supplemented calcium, vitamin D, and, in specific cases, bisphosphonate intake, as well as long-term prophylactic factor replacement therapy, were suggested as means of prevention of bone loss [2]. Furthermore, various inflammatory and angiogenetic processes have been implicated in early joint bleeding and in the pathogenesis of HJD. Achieving a deeper knowledge of HJD could potentially lead to earlier diagnosis and treatment in patients with haemophilia.

## HAEMOPHILIC ARTHROPATHY

### Structure of the Synovial Joint

**Figure 1:**
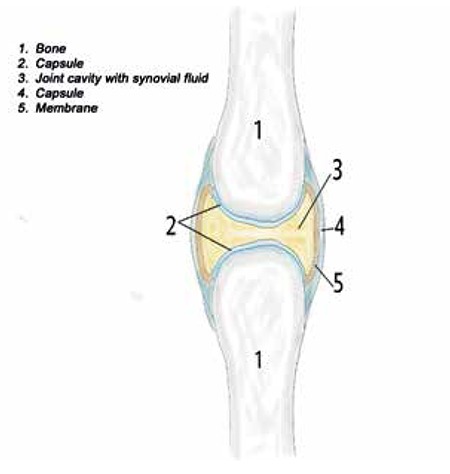
Synovial joint.

The synovial joint belongs to the group of joints that have to bear a great amount of movement. In such joints, the bony surfaces are covered with articular cartilage and are connected by ligaments. The joint may be divided by an articular disc or meniscus, which is continuous in the periphery with the fibrous capsule while its free surfaces are covered by the synovial membrane. Synovial joints facilitate movement by bringing articulating bones into contact (Figure 1).

The components of a synovial joint are the synovial cavity, which is the space between the bones filled with synovial fluid, and the articular capsule, which surrounds the joint and unites the articulating bones. The articular capsule also consists of two layers: the outer fibrous membrane, which may contain ligament, and the inner synovial membrane that secretes the lubricating synovial fluid. The bones of the synovial joint are covered by a layer of cartilage that functions to absorb tension and reduce friction during movement [3]. The articular capsule is highly innervated but is lacking blood vessels. The surrounding blood network provides the necessary nutritional supply [4].

HJD is the end result of a number of changes occurring in every component of the joint after repeated bleeding episodes. Bleeding into the synovial joint exposes synovial cells to blood, which is toxic for the joint. Morris et al. proposed that iron plays a substantial role in the development of haemophilic synovitis [5]. Studies by Wen et al. showed that iron is also involved in myelocytomatosis viral oncogene (MYC) and mouse double minute-2 (MDM2) homolog expression, which causes proliferation of the synovium and active inflammation [6]. Roosendaal and Lafeber observed that iron increases the expression of proinflammatory cytokines like interleukin-6 (IL-6), interleukin 1-β (IL-1β), and tumour necrosis factor-α (TNF-α) in synovial cells [7]. Histologically, it was shown that synovial inflammation incorporates three characteristics: 1) hypertrophy of the villi, 2) increased number of inflammatory cells, and 3) increased vascularisation [7,8,9].

### Synovial Angiogenesis

**Figure 2:**
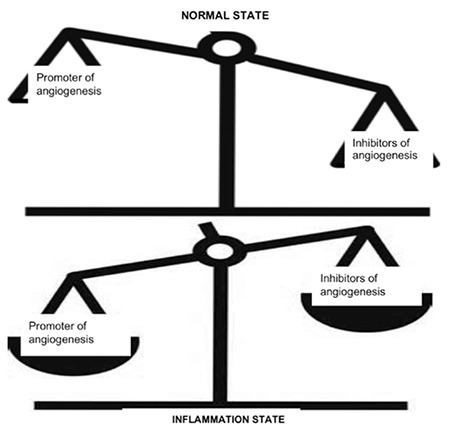
Angiogenesis in normal state and inflammation state.

Angiogenesis is a normal process during wound healing and embryogenesis. It is also considered part of the pathophysiologic mechanisms implicated in diseases like rheumatoid arthritis (RA), osteoarthritis, systemic lupus erythematosus, and carcinogenesis. It is regulated by various inducers and inhibitors. During inflammation, the inducers/promoters prevail over inhibitors (Figure 2).

Angiogenesis takes place mainly in the bone marrow and in vascular stem cells. Angiogenetic factors like vascular endothelial growth factor (VEGF), angiopoietin-1 (Ang-1), angiopoietin-2 (Ang-2), and fibroblast growth factor (FGF) participate in endothelial cell proliferation. 

In 2008, it was shown that mice with factor IX deficiency experienced delayed wound healing and increased wound angiogenesis along with subcutaneous haematoma formation days after induction of a wound. It was suggested that tissue damage induces coagulation and inflammation response [10].

For angiogenesis to be triggered, a series of events have to take place. Mediators from the synovium activate endothelial cells, which release proteolytic enzymes that act on the endothelial basement membrane and the perivascular extracellular matrix. The endothelial cells participate in the formation of primary sprouts. The lumen of the sprouts facilitate the formation of “capillary loops” followed by the synthesis of new basement membrane and new capillaries [11].

RA is among the inflammatory disorders in which increased angiogenesis is observed as well. By shedding light on the understanding of the relationship between angiogenesis and inflammatory arthritis, the role of new vessel formation in haemophilic arthropathy could be more easily comprehended.

Angiogenesis is induced by several conditions like hypoxia and injury where proangiogenic molecules are secreted by tissues.

Endothelial cell proliferation and migration are followed by capillary tube formation, deposition of basement membrane, and migration of smooth muscle cells. Anastomoses are created and the flow of blood is established [12].

In RA, inflammatory cells like macrophages, lymphocytes, mast cells, and fibroblasts, along with their soluble products including pro-inflammatory cytokines, TNF-α, IL-1, and IL-8, are all promoters of angiogenesis. One of the major endothelial growth factors found in the synovium of patients with RA is VEGF. In RA synovium, IL-1 and TNF-α facilitate the fibroblast expression of VEGF [13,14]. VEGF induces the endothelial cell decay-accelerating factor, which acts protectively for the cells against activated complement components and may regulate endothelial proliferation and angiogenesis [15].

Direct measurements conﬁrm that the intra-articular environment is hypoxic in inﬂammatory arthritis. Hypoxia is often a feature of inﬂammation and is a strong inducer of VEGF. Tissue hypoxia in the rheumatoid joint results in increased VEGF messenger ribonucleic acid (mRNA) stability [16] and enhanced VEGF gene transcription through the binding of hypoxia-inducible transcription factors such as hypoxia-induced factor-1 (HIF) and HIF-2 that are overexpressed in the synovial lining and stromal cells of RA patients relative to synovial tissues from individuals without arthritis [17].

Zimmermann in 1923 introduced the term ‘pericyte’ to describe a periendothelial support cell wrapped around the length of micro-vessels [18]. While it is known that pericytes are present in the microcirculation, their functional roles and importance in microvascular physiology has not been fully investigated. Recently pericytes have become a research area of growing interest as potential targets for pro- or antiangiogenic therapies. Vascular pericytes elongate around endothelial cells and their function is to assist in the regulation of vessel stabilisation and in endothelial cell proliferation. During angiogenesis, signals from the pericyte to the endothelial cell and vice versa are critical for the formation of the capillary sprout. Studies found that pericytes may have a leading role in newly formed capillaries. This implicates their role in endothelial cell guidance. During angiogenesis, pericytes are involved in recruitment and direct interaction with endothelial cells [19]. Moreover, they participate in the development of newly formed endothelial cells [20]. Pericyte development is usually controlled by platelet-derived growth factor-B (PDGF), secreted by endothelial cells [21].

It was found that pericytes are directly involved in the process of angiogenesis as increasing pericyte coverage via Ang-2 inhibition can potentially represent an antiangiogenic tumour therapy [22,23]. Advancing the understanding of pericytes and the ability to develop pericyte-related therapies is a challenging and very promising process.

## Angiogenesis and Haemophilia

**Figure 3:**
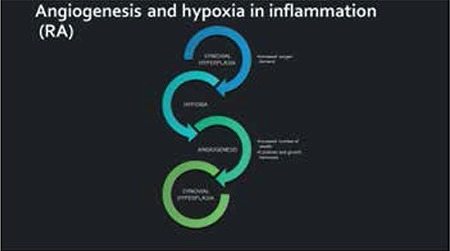
Angiogenesis and hypoxia in rheumatoid arthritis. RA: Rheumatoid arthritis.

Angiogenesis is a natural process considered as a physiologic response to inﬂammation, hypoxia, and malignancy. It may be mediated by various factors including growth factors, proinflammatory cytokines, chemokines, extracellular matrix molecules, matrix-degrading proteolytic enzymes, cellular adhesion molecules, and others [24]. One of the most important growth factors in terms of angiogenesis is VEGF. HIF acts by upregulating VEGF [17,25]. Furthermore, prostaglandin and nitric oxide act via VEGF in neovascularisation. There is an interaction between VEGF and Ang-1 that acts in favour of the newly formed vessels as a form of protection and stabilisation. On the contrary, Ang-2 antagonises Ang-1 and has a negative impact on neovascularisation (Figure 3).

VEGF and PDGF are involved in the development of inflammatory joint disease, potentially by favouring cytokine-related cartilage destruction and not synthetic cell responses associated with growth factor activity. Expression of VEGF is increased in individuals with inflammatory joint disease compared with normal controls. Among individuals with polyarthritis, concentrations of VEGF and its receptor, Ang-1, have been found to be related to inflammatory markers and bone destruction. Based on that, VEGF could play a role in haemophilic arthropathy [26].

There are a variety of factors that work in an inhibitory manner regarding formation of new vessels. Among them are cytokines like interferon-α (IFN-α), IFN-γ, IL-4, IL-12, and leukaemia inhibitory factor (LIF). Protease inhibitors like tissue inhibitor of metalloproteinase (TIMPS), plasminogen activator inhibitors (PAIs), and thrombospondin-1 inhibit capillary and new vessel formation [11,27].

Acharya et al. [28] observed that there is involvement of angiogenesis in the development of haemophilic synovitis. Sera from haemophilic subjects with joint arthropathy induced an angiogenic response in endothelial cells that was ceased by blocking VEGF while peripheral blood mononuclear cells from these subjects stimulated synovial cell proliferation, which was blocked by a humanised anti-VEGF antibody (bevacizumab). Human synovial cells, when incubated with haemophilic sera, could elicit upregulation of hypoxia-inducible factor-1A (HIF-1A) mRNA, indicating that hypoxia plays an important role in the neoangiogenesis process.

The onset of endothelial proliferation is based on the equilibrium between positive and negative regulators [29]. Once there is a stimulus from VEGF-A, endothelial cells became activated, proliferate, and form new vessels. However, these newly formed vessels are extremely sensitive and prone to bleeding. In order to protect them, PDGF stimulates pericytes, which migrate to the place of the angiogenesis and embrace the vessels with dendritic processes, forming a stabilising coat. The presence of pericytes is considered a sign of maturity.

In another study, Zetterberg et al. [30] observed that VEGF is increased in synovial tissue from haemophilic patients. In this study, synovial tissue was obtained when HJD was already established. The increased VEGF in synovial cells from these patients showed that HJD is characterised by active angiogenesis. Vessels from HJD and control synovial tissue were found to be covered by pericytes, indicating that most of the vessels were mature. Even though the number of patients with different stages of HJD in this study was too low to understand how angiogenesis develops over time in HJD, there were indications that end-stage HJD is characterised by a chronic proinﬂammatory, proangiogenic drive, while the vessel formation is relatively slow, permitting vessels to mature and develop pericyte “protection”.

Tattersall et al. [31] observed that macrophages enhance angiogenesis, increasing the number and length of endothelial sprouts, a property called “angiotrophism”. Polarising macrophages in a proinflammatory manner could increase their angiotrophic stimulation of vessel sprouting. This increase was found to be dependent on macrophage Notch signalling. JAG1 expression and Notch signalling are essential for the growth of both endothelial cells and pericytes.

In a recent study by Yi et al. [32], it was demonstrated that annexin a2 (Axna2) could promote the progression of RA. Axna2 plays an important role in pannus formation in RA. Cytological analysis showed that the Axna2/Axna2 receptor (Axna2/Axna2R) axis promoted new vessel formation by activation of the Hedgehog (HH) signalling pathway and increased the Patched (Ptc) and Smoothened (Smo) expression in order to upregulate the expression of the downstream metalloproteinases (MMPs), VEGF, and Ang-2. These results suggest that the effect of Axna2 might provide a new potential measure for treatment of RA and potentially HJD.

## NEW POTENTIAL THERAPIES: A STEP TO THE FUTURE

Cancer and inflammation research trials have been targeting angiogenic mediator and inhibitor pathways regarding the development of new therapeutic agents. There have been attempts to target VEGF by using synthetic VEGF and VEGFR inhibitors, anti-VEGF antibodies, and inhibitors of VEGF and VEGFR signalling, primarily in colorectal, lung, renal, and liver cancers. Bevacizumab, a human monoclonal antibody to VEGF, has been used in the treatment of various types of cancer [11]. Vatalanib, a VEGFR protein kinase inhibitor, inhibited knee arthritis in rabbits [33].

YC-1, a superoxide-sensitive stimulator of soluble guanylyl cyclase originally developed to treat hypertension and thrombosis, is also a HIF-1 inhibitor [34]. Microtubule destabilisers, such as 2-ME, as well as paclitaxel, an anticancer agent, also diminish HIF-1α expression and activity [35]. Infliximab treatment in combination with methotrexate reduced synovial VEGF expression and vascularity [36,37]. Anti-TNF therapy in arthritic patients reduced Ang-1 but stimulated Ang-2 expression [38]. Recently, the anti-IL-6 receptor antibody tocilizumab decreased serum levels of VEGF [39]. Thalidomide, currently used in multiple myeloma treatment but also tried in lupus and RA, is a potent TNF-α antagonist and angiogenesis inhibitor [27,40]. Thalidomide could suppress both synovitis and angiogenesis [27], suggesting that its antiangiogenic effects may be, in part, VEGF-independent. Fumagillin is a natural product of Aspergillus fumigatus. TNP-470 and PPI2458 are synthetic derivatives of fumagillin that inhibit methionine aminopeptidase-2, an enzyme involved in angiogenesis [41].

It would be extremely interesting to learn if some of these treatment options could be applied to haemophilia patients in the future and if they could have an impact on the development, progression, and treatment of synovitis and haemophilic joint arthropathy.

## CONCLUSION

Further studies will have to clarify the mechanisms and circumstances that may be responsible for modulating the contribution of angiogenesis to HJD. It is very intriguing to consider the possibility that angiogenetic factors may play a crucial role in the pathogenesis of arthropathy seen in patients suffering from haemophilia. Finally, the possibility that there may be potential markers enabling identification of the onset as well as the progression of haemophilic synovitis deserves further investigation.

## References

[1] Mannucci PM (2002). Haemophilia and related bleeding disorders: a story of dismay and success. Hematology Am Soc Hematol Educ Program.

[2] Anagnostis P, Karras SN, Goulis DG (2015). Bone disease in patients with haemophilia A and B -- where are we now?. Haemophilia.

[3] Boundless Anatomy and Physiology. Structure of Synovial Joints. Available online at.

[4] Boundless Anatomy and Physiology. Nerve and Blood Supply. Available online at.

[5] Morris CJ, Blake DR, Wainwright AC, Steven MM (1986). Relationship between iron deposits and tissue damage in the synovium: an ultrastructural study. Ann Rheum Dis.

[6] Wen FQ, Jabbar AA, Chen YX, Kazarian T, Patel DA, Valentino LA (2002). c-myc proto-oncogene expression in haemophilic synovitis: in vitro studies of the effects of iron and ceramide. Blood.

[7] Roosendaal G, Lafeber FP (2006). Pathogenesis of haemophilic arthropathy. Haemophilia.

[8] Acharya SS (2012). Exploration of the pathogenesis of haemophilic joint arthropathy: understanding implications for optimal clinical management. Br J Haematol.

[9] Øvlisen K, Kristensen AT, Jensen AL, Tranholm M (2009). IL-1β, IL-6, KCand MCP-1 are elevated in synovial fluid from haemophilic mice with experimentally induced haemarthrosis. Haemophilia.

[10] Hoffman M, Harger A, Lenkowski A, Hedner U, Roberts HR, Monroe DM (2006). Cutaneous wound healing is impaired in hemophilia B. Blood.

[11] Szekanecz Z, Koch AE (2007). Mechanism of disease: angiogenesis in inflammatory diseases. Nat Clin Pract Rheumatol.

[12] Desmoulière A, Redard M, Darby I, Gabbiani G (1995). Apoptosis mediates the decrease in cellularity during the transition between granuloma tissue and scar. Am J Pathol.

[13] Fava RA, Olsen NJ, Spencer-Green G, Yeo KT, Yeo TK, Berse B, Jackman RW, Senger DR, Dvorak HF, Brown LF (1994). Vascular permeability factor/endothelial growth factor (VPF/VEGF): accumulation and expression in human synovial fluids and rheumatoid synovial tissue. J Exp Med.

[14] Koch AE, Harlow LA, Haines GK, Amento EP, Unemori EN, Wong WL, Pope RM, Ferrara N (1994). Vascular endothelial growth factor. A cytokine modulating endothelial function in rheumatoid arthritis. J Immunol.

[15] Mason JC, Lidington EA, Yarwood H, Lublin DM, Haskard DO (2001). Induction of endothelial cell decay-accelerating factor by vascular endothelial growth factor: a mechanism for cytoprotection against complement-mediated injury during inflammatory angiogenesis. Arthritis Rheum.

[16] Richard DE, Berra E, Pouyssegur J (1999). Angiogenesis: how a tumor adapts to hypoxia. Biochem Biophys Res Commun.

[17] Giatromanolaki A, Sivridis E, Maltezos E, Athanassou N, Papazoglou D, Gatter KC, Harris AL, Koukourakis MI (2003). Upregulated hypoxia inducible factor-1α and -2α pathway in rheumatoid arthritis and osteoarthritis. Arthritis Res Ther.

[18] Zimmermann KW (1923). Der feinere Bau der Blutkapillaren. Z Anat.

[19] Gerhardt H, Betsholtz C (2003). Endothelial-pericyte interactions in angiogenesis. Cell Tissue Res.

[20] Ponce AM, Price RJ (2003). Angiogenic stimulus determines the positioning of pericytes within capillary sprouts in vivo. Microvasc Res.

[21] Hellström M, Kalén M, Lindahl P, Abramsson A, Betsholtz C (1999). Role of PDGF-B and PDGFR-β in recruitment of vascular smooth muscle cells and pericytes during embryonic blood vessel formation in the mouse. Development.

[22] Bergers G, Song S, Meyer-Morse N, Bergsland E, Hanahan D (2003). Benefits of targeting both pericytes and endothelial cells in the tumor vasculature with kinase inhibitors. J Clin Invest.

[23] Gerald D, Chintharlapalli S, Augustin HG, Benjamin LE (2013). Angiopoietin-2: an attractive target for improved antiangiogenic tumor therapy. Cancer Res.

[24] Szekanecz Z, Koch AE (2001). Chemokines and angiogenesis. Curr Opin Rheumatol.

[25] Taylor PC, Sivakumar B (2005). Hypoxia and angiogenesis in rheumatoid arthritis. Curr Opin Rheumatol.

[26] Valentino LA (2010). Blood-induced joint disease: the pathophysiology of hemophilic arthropathy. J Thromb Haemost.

[27] Lainer-Carr D, Brahn E (2007). Angiogenesis inhibition as a therapeutic approach for inflammatory synovitis. Nat Clin Pract Rheumatol.

[28] Acharya SS, Kaplan RN, Macdonald D, Fabiyi OT, DiMichele D, Lyden D (2011). Neoangiogenesis contributes to the development of hemophilic synovitis. Blood.

[29] Nadar SK, Karalis I, Al Yemeni E, Blann AD, Lip GY (2005). Plasma markers of angiogenesis in pregnancy induced hypertension. Thromb Haemost.

[30] Zetterberg E, Palmblad J, Wallensten R, Morfini M, Melchiorre D, Holmström M (2014). Angiogenesis is increased in advanced haemophilic joint disease and characterized by normal pericyte coverage. Eur J Haematol.

[31] Tattersall IW, Du J, Cong Z, Cho BS, Klein AM, Dieck CL, Chaudhri RA, Cuervo H, Herts JH, Kitajewski J (2016). In vitro modeling of endothelial interaction with macrophages and pericytes demonstrates Notch signaling function in the vascular microenvironment. Angiogenesis.

[32] Yi J, Zhu Y, Jia Y, Jiang H, Zheng X, Liu D, Gao S, Sun M, Hu B, Jiao B, Wang L, Wang K (2016). The annexin a2 promotes development in arthritis through neovascularization by amplification Hedgehog pathway. PLoS One.

[33] Grosios K, Wood J, Esser R, Raychaudhuri A, Dawson J (2004). Angiogenesis inhibition by the novel VEGF receptor tyrosine kinase inhibitor, PTK787/ZK222584, causes significant anti-arthritic effects in models of rheumatoid arthritis. Inflamm Res.

[34] Yeo EJ, Chun YS, Cho YS, Kim J, Lee JC, Kim MS, Park JW (2003). YC-1: a potential anticancer drug targeting hypoxia-inducible factor 1. J Natl Cancer Inst.

[35] Mabjeesh NJ, Escuin D, LaVallee TM, Pribluda VS, Swartz GM, Johnson MS, Willard MT, Zhong H, Simons JW, Giannakakou P (2003). 2ME2 inhibits tumor growth and angiogenesis by disrupting microtubules and dysregulating HIF. Cancer Cell.

[36] Veale DJ, Fearon U (2006). Inhibition of angiogenic pathways in rheumatoid arthritis: potential for therapeutic targeting. Best Pract Res Clin Rheumatol.

[37] Goedkoop AY, Kraan MC, Picavet DI, Rie MA, Teunissen MB, Bos JD, Tak PP (2004). Deactivation of endothelium and reduction in angiogenesis in psoriatic skin and synovium by low dose infliximab therapy in combination with stable methotrexate therapy: a prospective single-centre study. Arthritis Res Ther.

[38] Markham T, Mullan R, Golden-Mason L, Rogers S, Bresnihan B, Fitzgerald O, Fearon U, Veale DJ (2006). Resolution of endothelial activation and down-regulation of Tie2 receptor in psoriatic skin after infliximab therapy. J Am Acad Dermatol.

[39] Nakahara H, Song J, Sugimoto M, Hagihara K, Kishimoto T, Yoshizaki K, Nishimoto N (2003). Anti-interleukin-6 receptor antibody therapy reduces vascular endothelial growth factor production in rheumatoid arthritis. Arthritis Rheum.

[40] D’Amato RJ, Loughnan MS, Flynn E, Folkman J (1994). Thalidomide is an inhibitor of angiogenesis. Proc Natl Acad Sci U S A.

[41] Ingber D, Fujita T, Kishimoto S, Sudo K, Kanamaru T, Brem H, Folkman J (1990). Synthetic analogues of fumagillin that inhibit angiogenesis and suppress tumour growth. Nature.

